# Effectiveness of different antithrombotic agents in combination with tranexamic acid for venous thromboembolism prophylaxis and blood management after total knee replacement: a prospective randomized study

**DOI:** 10.1186/s12891-022-06117-8

**Published:** 2023-01-04

**Authors:** Li-Bo Zhou, Chao-Chao Wang, Lan-Tao Zhang, Tao Wu, Guo-Qiu Zhang

**Affiliations:** 1 Department of Graduate School, Qinghai University, No. 251 Ningda Road, Chengbei District, Xining, 810016 Qinghai Province China; 2 Department of Bone and Joint Surgery, Affiliated Hospital of Qinghai University, No. 29 Tongren Road, Chengxi District, Xining, 810012 Qinghai Province China

**Keywords:** Total knee arthroplasty, Tranexamic acid, Rivaroxaban, Dalteparin sodium; Aspirin, Venous thromboembolism

## Abstract

**Background:**

Tranexamic acid (TXA) has been widely applied in total knee arthroplasty (TKA) to significantly reduce perioperative blood loss and improve knee function recovery in patients after surgery. The choice of antithrombotic agents for venous thromboembolism (VTE) prevention after TKA is controversial. Therefore, this study aimed to compare the effects of different antithrombotic agents on patients after primary unilateral TKA in the context of applied TXA.

**Methods:**

A total of 180 patients undergoing primary unilateral TKA from October 2020 to December 2021 were included in this study. All patients were given an intraoperative drip of 60 mg/kg TXA. Thereafter, patients were divided into three groups (*n* = 60 each). Baseline data were comparable among the three groups. The average follow-up time was 3.02 ± 0.09 months. Group 1 enrolled patients receiving oral rivaroxaban (RA) at 10 mg, Group 2 included patients who received subcutaneous Dalteparin sodium at 2500 IU, while Group 3 included patients taking oral aspirin (ASA) at 100 mg. Patients in all the three groups received treatment once a day for 30 days at 12 h postoperatively. The primary outcomes in this study were post-treatment drainage volume and thrombotic complication rate. The secondary outcomes included hematologic parameters, transfusion rate, intraoperative blood loss, total blood loss (TBL), and bleeding complication rate.

**Results:**

The average drainage volume after treatment was significantly lower in Group 3 than in Group 1 and Group 2 (205.2 ± 69.0 vs 243.4 ± 72.5 vs 295.4 ± 72.5 ml, *P* < 0.001), and there was a significant difference between Group 1 and Group 2 (243.4 ± 72.5 mL vs 295.4 ± 72.5 mL, *P* < 0.001). The blood transfusion rate of Group 2 dramatically increased compared with Group 1 and Group 3 (20.0% vs 6.7% vs 5.0%, *P* = 0.01). The bleeding complication rate in Group 1 apparently increased relative to Group 2 and Group 3 (26.7% vs 10.0% vs 8.3%, *P* = 0.008). Besides, there was no significant difference in the thrombotic complication rate among the three groups.

**Conclusion:**

Under the background of TXA application, ASA, RA, and Dalteparin sodium were all effective on preventing VTE after TKA. In addition, ASA effectively reduced post-treatment Hemoglobin (Hb) loss, drainage volume, TBL, transfusion rate, and bleeding complications compared with RA and Dalteparin sodium.

**Trial registration:**

The trial was registered at the Chinese Clinical Trial Registry (ChiCTR2200060169). Date of Registration: 21/05/2022.

## Background

Total knee arthroplasty (TKA) is the treatment of choice for end-stage osteoarthritis, which can reduce pain and improve knee function [1]. Perioperative blood loss, one of the major complications following TKA, can exceed 1000 mL [2]. The postoperative allogeneic blood transfusion rate is 78.4% [3]. Notably, massive blood loss will increase the risk of perioperative complications and the economic burden on patients [4]. Intravenous tranexamic acid (TXA) proved to be effective on TKA in the perioperative period, which can reduce blood loss, blood transfusion rate, swelling, and ecchymosis [5–7]. In the perioperative period of TKA, patients are at a high risk of venous thromboembolism (VTE) due to the application of tourniquets, bone cement, endothelial injury and reduced postoperative activity [8]. Therefore, how to reduce the risk of VTE while achieving anti-fibrinolysis has become a hot topic in clinical research. Currently, the use of anticoagulants or antiplatelet agents has been recommended by various guidelines to prevent thrombosis after TKA, but it remains unclear which agent has the best risk-benefit profile [9–11]. Rivaroxaban (RA) is effective on reducing the incidence of deep venous thrombosis (DVT) and pulmonary embolism (PE) after TKA, with no need to closely monitor the coagulation parameters. However, a higher incidence of knee swelling, ecchymosis and wound complications in patients has been demonstrated [12]. Dapsigargin sodium is a low molecular weight heparin (LMWH), which has the characteristics of good anticoagulant effect, long half-life and small difference in anticoagulant dosage among patients, as a result, it has been extensively applied in clinical practice [13]. Aspirin (ASA) displays unique advantages due to its low price, administration safety, and ease of use. However, there is no evidence about whether ASA can replace other anticoagulants for thromboprophylaxis after TKA [14, 15]. In the present prospective randomized study, the primary outcomes were post-treatment drainage volume and thrombotic complication rates (including VTE rates and PE rates). The secondary outcomes included hematologic parameters, blood transfusion rate, intraoperative blood loss, total blood loss (TBL), and bleeding complication rates (including incision bleeding rates, subcutaneous ecchymosis rates, and gastrointestinal bleeding rates). Of them, hematological parameters included Hb level, platelet count, prothrombin time (PT), activated partial thromboplastin time (APTT), and patient’s blood volume (PBV). Therefore, it is hypothesized that, compared with TXA plus RA/Dalteparin, TXA combined with ASA can significantly reduce blood loss, blood transfusion rate, and bleeding complication rate in TKA after treatment, and all the three antithrombotic agents can effectively prevent VTE.

## Methods

### Patient inclusion and exclusion criteria

**Fig. 1:**
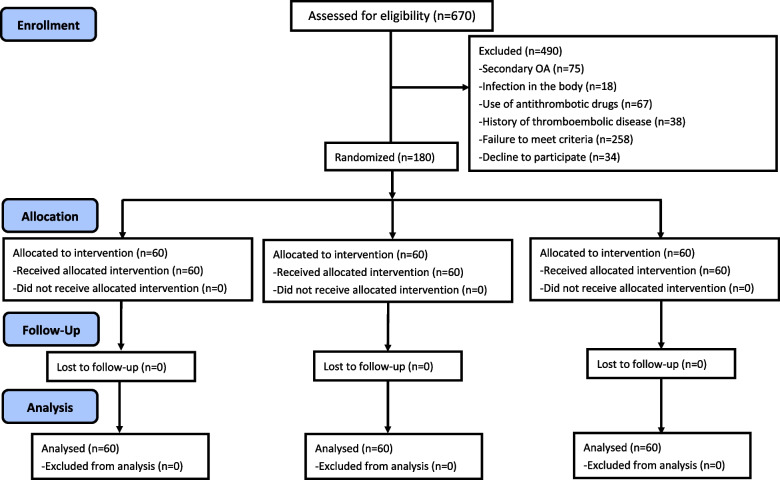
CONSORT flow diagram for this clinical trial

The present study prospectively designed and collected clinical data from patients undergoing TKA at our center from October 2020 to December 2021. Finally, a total of 180 patients undergoing primary unilateral TKA were included into this work (Fig. 1). The average follow-up period was 3.02 ± 0.09 months, with no loss to follow-up. The patient inclusion criteria were as follows, (1) those aged 55–80 years; (2) patients diagnosed with knee osteoarthritis according to the Kellgren-Lawrence grading system (greater than or equal to grade III) [16]; and (3) those who were willing to undergo primary unilateral TKA. Patients conforming to the following exclusion criteria were eliminated out of this work: (1) those with secondary osteoarthritis, such as post-traumatic arthritis, rheumatoid arthritis and gouty arthritis; (2) those with systemic or local infection; (3) those with blood system diseases; (4) those with previous or current use of antithrombotic drugs; (5) those with a previous history of thrombosis or thrombosis discovered on color Doppler ultrasound of both lower extremities; (6) those developing high-risk cardiovascular disease (CVD) with thromboses, including cerebral infarction, myocardial infarction, atrial fibrillation, heart failure and post-stenting; (7) those taking non-steroidal anti-inflammatory drugs (NSAIDs); and (8) those with a history of epilepsy or severe liver and kidney insufficiency. This study was conducted at the Department of Joint Surgery, Qinghai University Affiliated Medical School, and registered at the Chinese Clinical Trials Registry (ChiCTR2200060169). Our study protocols were approved by the Ethics Committee of Clinical School of Qinghai University (P-SL-2022-039), and all patients provided the informed consents for participation. The authors confirmed that all the ongoing and related trials for this drug/intervention are registered and adhere to the CONSORT guidelines.

### Randomization and blinding

All patients were randomized into three groups (Group 1, Group 2, and Group 3) according to a computerized randomization list generated by Randomization.com. The randomization was prepared by a statistician who was not involved in this clinical trial. Besides, the randomization assignments were placed within sequentially numbered opaque sealed envelopes in the custody of a certified study pharmacist. At the end of the procedure, the envelopes were opened, and the corresponding medications were handled by an investigator who was blinded to the patient’s care. Patients, trial participants, outcome assessors, and data collectors were blinded to allocation.

### Interventions

Group 1: Patients were given RA (10 mg, Lierban, Shanghai Huilun Jiangsu Pharmaceutical Co, China) orally 12 h postoperatively and then once daily for 30 days [17–19]. Group 2: Patients were administered with Dalteparin sodium (2500 IU, Qianhongyida, Changzhou Qianhong Biochemical Pharmaceutical Co, China) by subcutaneous injection 12 h postoperatively and then once daily for 30 days [20]. Group 3: Patients were given ASA (100 mg, Bayaspirin, Bayer, Germany) orally 12 h postoperatively and then once daily for 30 days [21, 22].

### Surgical methods and postoperative treatment

All procedures were performed by the same orthopedic surgeon (senior level). All patients were given 60 mg/kg TXA intravenously 5 min prior to prosthesis placement [23–25]. The combined lumbar and rigid anesthesia was chosen, and a medial parapatellar approach was selected with a median skin incision made anterior to the knee under the control of a balloon tourniquet. TKA was subsequently performed according to the routine procedure, one drainage tube was placed, then the incision was closed, and pressure bandaging was completed. Prophylactic antibiotics were administered intravenously 30 min before excision, and antibiotics were applied continuously for 7 days postoperatively.

Patients’ vital signs and state of consciousness were closely monitored, meanwhile the blood supply and drainage volume of the affected limb were also observed. The drainage tube was removed 24 h after surgery and the drainage volume was recorded. After recovery from anaesthesia, each patient was instructed to lie flat and keep the affected limb straight for ankle pump exercises. Compression bandaging of the affected limb and ice packs were recommended 24 h after surgery. Multimodal analgesia using analgesic pumps combined with NSAIDs was recommended. On the second day after surgery, patients were encouraged to perform active and passive knee flexion and extension exercises under the help of the surgeon and walk with the help of a walker.

According to the clinical transfusion guidelines released by the Chinese Ministry of Health, allogeneic blood transfusions are given if the Hb is < 70 g/L in asymptomatic patients or 70–100 g/L in symptomatic patients (namely, fatigue, poor appetite, anemia, or myocardial ischemia). An attending physician who was not involved in this study were responsible for the decision-making of blood transfusion [26].

### Outcome measures

Demographic parameters including age, gender, side, and body mass index (BMI) were compared.

Primary outcomes included post-treatment drainage volume and thrombotic complication rate. Post-treatment drainage volume was defined as the total amount of fluid drained from the drainage tube 24 h post-treatment (including the 24th h). The thrombotic complication rate was determined by dividing the number of patients with thrombosis in each group by the total number of patients. Patients underwent Doppler ultrasonography prior to TKA to assess the presence or absence of DVT. Moreover, patients received lower extremity arteriovenous ultrasound on days 3, 7, 30, 60 and 90 postoperatively, and DVT was defined if the results were positive. If PE-associated symptoms were found, the diagnosis of PE was confirmed by the positive results of CT pulmonary angiography (CTPA).

Secondary outcomes included hematologic parameters, blood transfusion rate, TBL, intraoperative blood loss, and bleeding complication rate. Hematological parameters included pre-treatment and post-treatment hematological parameters. Of them, pre-treatment hematological parameters included Hb level, platelet count, PT, APTT, and PBV before treatment. The PBV before treatment was assessed according to the formula proposed by Nadler et al. [27]:

$$\textrm{PBV}\ \textrm{before}\ \textrm{treatment}\ \left(\textrm{m}\textrm{L}\right)=\textrm{k}1\times \textrm{height}\ \left(\textrm{m}\right)+\textrm{k}2\times \textrm{weight}\ \left(\textrm{kg}\right)+\textrm{k}3;\textrm{k}1=0.3669,\textrm{k}2=0.03219,\textrm{and}\ \textrm{k}3=0.6041\ \textrm{for}\ \textrm{men};\textrm{k}1=0.3561,\textrm{k}2=0.03308,\textrm{and}\ \textrm{k}3=0.1833\ \textrm{for}\ \textrm{women}.$$

Post-treatment hematological parameters included Hb level, platelet count, PT and APTT on days 1, 3 and 5 after treatment. Platelet count, PT and APTT were collected from patients on days 1, 3 and 5 after treatment. As a result, there was no significant difference in platelet count, PT and APTT between the three groups. Therefore, platelet count, PT and APTT on day 3 after treatment were compared with those before treatment. The blood transfusion rate was calculated by dividing the number of patients receiving blood transfusions in each group by the total number of patients. Meanwhile, TBL was assessed according to the formula put forward by Gross et al. [28]:

$$\textrm{TBL}=\textrm{PBV}\times \left(\ \textrm{Hct}\ \textrm{pre}-\textrm{Hct}\ \textrm{post}\ \right)/\textrm{Hct}\ \textrm{ave}$$

Where PBV indicates patient’s blood volume, Hct pre stands for the initial preoperative hematocrit level, Hct post represents the lowest postoperative hematocrit level during hospitalization or the lowest postoperative hematocrit prior to blood transfusion, and Hct ave. indicates the average of Hct pre and Hct post. If a reinfusion or an allogenic transfusion was conducted, the volume transfused was added when calculating TBL.

Intraoperative blood loss was determined by the net increase in gauze used intraoperatively plus the amount of fluid in the drainage bottle at the end of the procedure minus the amount of fluid used for flushing. Bleeding complications included bleeding from the incision, subcutaneous petechiae, and bleeding from other parts of the body.

### Sample size and statistical analysis

According to the results of preliminary research and follow-up of some people in our department, the predictive effective rate of RA was 98%, that of ASA was 78%, and that of Dalteparin sodium was 87%, with α = 0.05 and 1- β = 0.8. Thereafter, the sample size was estimated by SPSS software (version 26.0, IBM Corp., Armonk, USA), and the final calculation result was *n* = 156. Taking loss to follow-up in the follow-up period into consideration, the minimum sample size required was n ≈ 165 if the loss to follow-up rate was 5%. Assuming that the sample sizes of the three groups were the same, then at least 55 subjects were needed in each treatment group. Based on the above sample size estimation results, a total of 180 patients were collected and divided into three groups, including Group 1, Group 2, and Group 3, with 60 cases each. Therefore, the sample size collected met the minimum sample size requirements for the study.

SPSS software was employed for statistical analysis. The normally-distributed measurement data were represented by mean ± standard deviation (SD). One-way analysis of variance (ANOVA) was utilized to compare three groups, while two-way repeated measurement ANOVA was used to compare different time points of each group, one-way repeated measurement ANOVA was adopted for comparison of indicators at different time points in the same group, and pairwise comparison was completed with the LSD method. The classification and counting data were expressed as %, and comparison between groups was expressed as χ^2^ inspection at the inspection level of α = 0.05 (two-sided).

## Results

### Patient demographics

There was no statistically significant difference in age, gender, side, or BMI between the three groups (*P =* n.s.) (Table 1).

**Table 1:** Patient demographics

Group	n	Age (years, SD)	Gender (n, M / F)	Side (n, L / R)	BMI (kg / m^2^, SD)
Group 1	60	64.8 ± 7.2	27 / 33	26 / 34	25.4 ± 4.2
Group 2	60	64.1 ± 6.7	29 / 31	25 / 35	24.9 ± 3.8
Group 3	60	66.4 ± 7.6	26 / 34	28 / 32	25.6 ± 3.3
*F/χ*^*2*^	–	1.5	0.3	0.3	0.4
*P*	–	0.2	0.8	0.8	0.6

*SD* Standard deviation, *M* Male, *F* Female, *L* Left, *R* Right, *BMI* Body mass index

### Hematologic parameters and blood transfusion rate

There was no significant difference in Hb levels among the three groups before treatment (*P* = 0.3). The Hb levels in Group 3 was higher than those in Group 1 and Group 2 on days 1, 3, and 5 after treatment (*P* < 0.001). The Hb levels in Group 1 dramatically increased relative to those in Group 2 on days 1, 3, and 5 after treatment (*P* < 0.001) (Table 2).

**Table 2:** Hb levels compared among three groups (g / L)

Group	n	Before treatment	Day 1 after treatment	Day 3 after treatment	Day 5 after treatment	Between groups	Point of time	Inter group· time point
Group 1	60	139.2 ± 11.2	117.5 ± 11.9	93.3 ± 11.8^*^	96.7 ± 11.7^*▲^	F = 20.0 *P* < 0.001	F = 659.5 P < 0.001	F = 7.3 P < 0.001
Group 2	60	141.3 ± 11.7	111.3 ± 13.9	87.2 ± 10.2^*^	90.6 ± 11.0^*▲^			
Group 3	60	138.0 ± 13.0	124.1 ± 14.0	99.3 ± 11.7^*^	102.7 ± 12.4^*▲^			
*F*	–	1.1	13.9	17.2	15.7			
*P*	–	0.3	< 0.001	< 0.001	< 0.001			

* Compared with day 1 after treatment, *P* < 0.001; ▲ Compared with day 3 after treatment, *P* < 0.001

Differences in platelet count, PT and APTT were not significant among the three groups before treatment (*P* = n.s). Compared with those before treatment, platelet count, PT and APTT of the three groups on the third day after treatment were of no significant difference (*P* = n.s). On day 3 after treatment, there was no significant difference in platelet count, PT or APTT among the three groups (*P* = n.s) (Table 3).

**Table 3:** Platelet count, PT, and APTT of the three groups before and after treatment

Group	n	After treatment			Before treatment		
		Platelet count (× 10^9^ / L, SD)	PT (S, SD)	APTT (S, SD)	Platelet count (×10^9^ / L, SD)	PT (S, SD)	APTT (S, SD)
Group 1	60	237.2 ± 52.2	11.2 ± 0.7	28.4 ± 3.4	227.6 ± 44.8^*^	11.0 ± 0.6^▲^	28.0 ± 4.2^△^
Group 2	60	243.1 ± 43.5	11.3 ± 0.8	27.7 ± 3.6	241.5 ± 46.7^*^	11.2 ± 0.8^▲^	27.4 ± 3.6^△^
Group 3	60	228.3 ± 39.2	11.4 ± 0.4	28.2 ± 3.3	235.7 ± 49.5^*^	11.2 ± 0.7^▲^	28.3 ± 3.4^△^
F	–	1.6	1.2	0.6	1.3	1.2	0.8
*P*	–	0.2	0.2	0.5	0.2	0.3	0.4

* Compared with platelet count before treatment, P = n.s; ▲ Compared with PT before treatment, P = n.s; △ Compared with APTT before treatment, P = n.s; *PT* Prothrombin time, *APTT* Activated partial thromboplastin time, *S* Second, *SD* Standard deviation

There was no significant difference in PBV between the three groups before treatment (*P* = 0.9). Group 2 reported a significantly increased blood transfusion rate compared with Group 1 and Group 3 after treatment (*P* = 0.01) (Table 4).

**Table 4:** PBV after treatment and blood transfusion rate before treatment of the three groups

Group	n	PBV after treatment (ml, SD)	Blood transfusion rate before treatment (n, %)
Group 1	60	4381.9 ± 838.1	4 (6.7)
Group 2	60	4376.2 ± 759.2	12 (20.0)^*^
Group 3	60	4429.4 ± 711.4	3 (5.0)^▲^
*F/χ*^*2*^	–	0.0	8.5
*P*	–	0.9	0.01

* Compared with Group 1, *P* = 0.01; ▲ Compared with Group 2, *P* = 0.01; *SD* Standard deviation

### Blood loss parameters

There was no significant difference in intraoperative blood loss among the three groups (*P* = 0.5). Besides, drainage volume and TBL after treatment were significantly lower in Group 3 than in Group 1 and Group 2 (*P* < 0.001), while those in Group 1 were remarkably lower than those in Group 2 after treatment (*P* < 0.001) (Table 5).

**Table 5:** Blood loss parameters among the three groups (ml, SD)

Group	n	Intraoperative blood loss	Drainage volume after treatment	TBL
Group 1	60	269.7 ± 88.0	243.4 ± 72.5	902.1 ± 129.5
Group 2	60	274.1 ± 87.3	295.4 ± 72.5^*^	959.0 ± 131.9^*^
Group 3	60	256.4 ± 83.5	205.2 ± 69.0^*▲^	833.2 ± 115.0^*▲^
F	–	0.6	24.1	15.0
*P*	–	0.5	< 0.001	< 0.001

* Compared with Group 1, *P* < 0.001; ▲ Compared with Group 2, *P* < 0.001; *SD* Standard deviation

### Bleeding complications and thrombotic complications

The bleeding complication rate in Group 1 dramatically increased compared with those in Group 2 and Group 3 (*P* = 0.008).

All patients with bleeding complications had gradual resolution of symptoms after discontinuation of RA/Dalteparin sodium/ASA (Table 6).

**Table 6:** Bleeding complications among the three groups (n, %)

Group	n	Incision bleeding	Subcutaneous ecchymosis	Gastrointestinal bleeding	Total incidence
Group 1	60	5 (8.3)	10 (16.7)	1 (1.7)	16 (26.7)
Group 2	60	2 (3.3)	4 (6.7)	0 (0.0)	6 (10.0)*
Group 3	60	1 (1.7)	3 (5.0)	1 (1.7)	5 (8.3)*
*χ*^*2*^	–	–	–	–	9.6
*P*	–	–	–	–	0.008

Compared with Group 1, *P* = 0.008

Difference in the thrombotic complication rate between the three groups of patients was not significant (*P* = 0.6). No PE was reported in any of the three groups.

Patients who developed thrombotic complications were invited for vascular surgery consultation and continued the use of RA/Dalteparin sodium/ASA. One patient developing left common femoral vein and superficial femoral vein thrombosis was given placement of an inferior vena cava filter and continued the anticoagulation therapy. After the thrombus gradually disappeared, the inferior vena cava filter was removed (Table 7).

**Table 7:** Thrombotic complications among the three groups (n, %)

Group	n	VTE between the muscles of lower limbs	VTE in the left common femoral vein and superficial femoral vein	Total incidence
Group 1	60	4 (6.7)	0 (0.0)	4 (6.7)
Group 2	60	4 (6.7)	1 (1.7)	5 (8.3)
Group 3	60	7 (11.7)	0 (0.0)	7 (11.7)
*χ*^*2*^	–	–	–	0.9
*P*	–	–	–	0.6

## Discussion

The most important finding of this study was that ASA, RA, and Dalteparin sodium were all effective on preventing VTE after TKA in the context of TXA application. In addition, ASA dramatically reduced post-treatment Hb loss, drainage volume, TBL, blood transfusion rate, and bleeding complication rate compared with RA and Dalteparin sodium. It is reported that if VTE prevention is not performed in major orthopedic surgery, the natural incidence rate is as high as 40–60%, by contrast, the incidence rate of symptomatic VTE in 3 months is only 1.3–10% through routine VTE prevention [29]. In this study, there was no significant difference in the incidence of thrombosis among the three groups, consistent with the results of several recent meta-analyses and large studies. As reported in the meta-analysis conducted by Matharu et al. [30], there was no significant difference in the application of ASA, LMWH, or RA in the prevention of VTE and adverse events after TKA. According to the retrospective study conducted by Hood et al. [31] (*n* = 41,537 cases), difference in the application of ASA, LMWH, and Xa inhibitor (Xa I) in preventing VTE after TKA was not significant. In addition, Anderson et al. [32] carried out a multicenter double-blinded randomized controlled trial enrolling 3424 patients with THA and TKA. The patients received RA (10 mg) orally once a day until the 5th day after surgery, and were later randomly assigned to TKA group (another 9 days of RA or ASA use at 81 mg/d) or THA group (another 30 days of RA or ASA use at 81 mg/d) for thrombosis prevention. Their results revealed no significant difference between ASA and RA in prolonging or preventing symptomatic VTE in patients receiving RA prevention for 5 days after THA and TKA. A retrospective study by Bala et al. [12] detected differences in the incidence rates of DVT and PE after 2-week and 90-day treatment with ASA, warfarin, enoxaparin, and Xa I after TKA (*P* < 0.01). In terms of DVT incidence, Xa I had the lowest rate, followed by ASA, enoxaparin, and warfarin. With regard to PE incidence, Xa I had the lowest incidence, followed by enoxaparin, ASA, and warfarin.

In this study, ASA evidently reduced post-treatment Hb loss, drainage volume, TBL, blood transfusion rate, and bleeding complication rate compared with RA and Dalteparin sodium. Bala et al. [12] compared the effectiveness of ASA, enoxaparin, Xa I, and warfarin on the prevention of venous thrombosis after TKA. According to their results, there was a difference in the incidence of anemia among patients 90 days postoperatively (*P* < 0.01). Typically, the incidence of anemia was the lowest in patients treated with ASA postoperatively (19%), followed by warfarin (22%), enoxaparin (23%), and Xa I (23%). Moreover, the blood transfusion rate was significantly different among patients 90 days postoperatively (*P* < 0.01). The lowest blood transfusion rate was seen in ASA (7%), followed by Xa I (9%), warfarin (12%), and enoxaparin (13%). Furthermore, Richardson et al. [33] found that 1.82% of 30,813 patients were diagnosed with VTE. Using ASA as the baseline, the risk of blood transfusion was significantly higher for LMWH (OR 1.56) and sulforaphane (OR 1.84). Similar results were reported in a study by Radzak et al. [34], where ASA was as effective as Lovenox on preventing VTE in patients with TKA, and the use of Lovenox apparently increased the probability of blood transfusion after surgery. Therefore, when using LMWH for thrombosis prevention, clinicians should closely monitor the changes in Hb decline and post-treatment drainage volume in patients, promptly replenish blood volume according to the patient condition, and provide other symptomatic supportive treatments. As indicated in the meta-analysis carried out by Marannes et al. [35], Xa I was more effective than ASA, LMWH, and warfarin on preventing VTE after TKA, but it was associated with a significantly increased bleeding complication rate. Zou et al. [18] compared the efficacy and safety of ASA, RA, and LMWH in the prevention of DVT after TKA. As a result, RA had a stronger anticoagulant effect but led to significantly increased postoperative blood loss and wound complications. In addition, Brimmo et al. [36] discovered that, compared with patients receiving other antithrombotic agents, those receiving RA after TKA had a significantly higher incidence of early deep surgical site infections. Similarly, it has been shown that the application of ASA in preventing VTE after TKA can reduce the incidence of periprosthetic infections [37]. Although infection was not detected in our patients, a significantly higher rate of bleeding-related events was observed in RA group, consistent with the above-mentioned literature reports. Therefore, when using RA for anticoagulation, clinicians should closely monitor the patient’s bleeding risk and wound healing, and give symptomatic supportive treatment. Some studies have indicated that ASA is as effective as or more effective than other anticoagulants even in the setting of high-risk VTE, such as in concurrent bilateral TKA or high-risk patient groups [38, 39]. In conclusion, it is necessary to assess the optimal balance between expected risks and benefits for each patient, so as to guide the selection of thromboprophylaxis agents.

Certain limitations should be noted in this study. First, this study had a small sample size, and the findings might be somewhat biased. Second, patients with severe comorbidities were excluded, while the relatively healthy patients were included in this study. The healthy study population might raise a concern of representing the majority of TKA patients regarding the application of antithrombotic agents. However, a randomized controlled trial was carried out to avoid selection bias, and the exclusion criteria seemed reasonable considering the potential adverse effects of antithrombotic agents.

## Conclusions

Collectively, our results suggest that ASA, RA and dalteparin sodium are all effective on preventing VTE after TKA. In addition, compared with RA and dalteparin sodium, ASA significantly reduces post-treatment Hb loss, drainage volume, TBL, blood transfusion rate, and bleeding complication rate. Aspirin application may be a safe and effective modality for VTE prophylaxis following TKA in the future.

## Data Availability

The datasets generated and/or analyzed in the current study are not publicly available because the participants did not consent to release of their data, but they are available from the corresponding author on reasonable request.

## Abbreviations

OA: Osteoarthritis
TKA: Total knee arthroplasty
TXA: Tranexamic acid
VTE: Venous thromboembolic event
DVT: Deep venous thrombosis
PE: Pulmonary embolism
RA: Rivaroxaban
ASA: Aspirin
TBL: Total blood loss
LMWH: Low molecular weight heparins
Hb: Hemoglobin
CVD: Cardiovascular disease
NSAIDs: Nonsteroidal anti-inflammatory drugs
BMI: Body mass index
SD: Standard deviation
PT: Prothrombin time
APTT: Activated partial thromboplastin time
CTPA: CT pulmonary angiography
Xa I: Xa inhibitor
